# SERS Determination of Oxidative Stress Markers in Saliva Using Substrates with Silver Nanoparticle-Decorated Silicon Nanowires

**DOI:** 10.3390/bios13020273

**Published:** 2023-02-14

**Authors:** Anastasia Kanioura, Georgia Geka, Ioannis Kochylas, Vlassis Likodimos, Spiros Gardelis, Anastasios Dimitriou, Nikolaos Papanikolaou, Sotirios Kakabakos, Panagiota Petrou

**Affiliations:** 1 Immunoassays/Immunosensors Laboratory, Institute of Nuclear & Radiological Sciences & Technology, Energy & Safety, NCSR “Demokritos”, 15341 Aghia Paraskevi, Greece; 2 Section of Condensed Matter Physics, Department of Physics, National and Kapodistrian University of Athens, University Campus, 15784 Athens, Greece; 3 Institute of Nanoscience & Nanotechnology, NCSR “Demokritos”, 15341 Aghia Paraskevi, Greece

**Keywords:** surface-enhanced Raman spectroscopy, oxidative stress, glutathione, crystal violet, malondialdehyde, thiobarbituric acid, saliva

## Abstract

Glutathione and malondialdehyde are two compounds commonly used to evaluate the oxidative stress status of an organism. Although their determination is usually performed in blood serum, saliva is gaining ground as the biological fluid of choice for oxidative stress determination at the point of need. For this purpose, surface-enhanced Raman spectroscopy (SERS), which is a highly sensitive method for the detection of biomolecules, could offer additional advantages regarding the analysis of biological fluids at the point of need. In this work, silicon nanowires decorated with silver nanoparticles made by metal-assisted chemical etching were evaluated as substrates for the SERS determination of glutathione and malondialdehyde in water and saliva. In particular, glutathione was determined by monitoring the reduction in the Raman signal obtained from substrates modified with crystal violet upon incubation with aqueous glutathione solutions. On the other hand, malondialdehyde was detected after a reaction with thiobarbituric acid to produce a derivative with a strong Raman signal. The detection limits achieved after optimization of several assay parameters were 50 and 3.2 nM for aqueous solutions of glutathione and malondialdehyde, respectively. In artificial saliva, however, the detection limits were 2.0 and 0.32 μM for glutathione and malondialdehyde, respectively, which are, nonetheless, adequate for the determination of these two markers in saliva.

## 1. Introduction

Oxidative stress is a condition characterized by an imbalance between the production of reactive oxygen species in the cells of a living organism and their ability to deactivate these highly reactive compounds [1]. The accumulation of reactive oxygen species in the cells and tissues could have detrimental effects on human health since it affects almost all cellular functions, leading to severe pathological situations such as metabolic disorders, cancer, atherosclerosis, and cardiovascular diseases [1]. To determine the oxidative stress status of an organism, several markers are used in the clinical practice, including either compounds produced due to excessive ROS presence, such as malondialdehyde and advanced oxidation protein products, or compounds with antioxidant activity such as glutathione, superoxide dismutase, and catalase [2].

Glutathione (GSH) is a tripeptide composed of glutamate, cysteine, and glycine, and it is considered one of the most abundant antioxidants in living organisms responsible for maintaining redox homeostasis through its conversion to an oxidized disulfide form [3]. On the other hand, malondialdehyde (MDA) is a product of lipid peroxidation caused by oxygen free radicals [4]. MDA binds to proteins and nucleic acids, disrupting protein synthesis and DNA transcription and affecting mitochondria and cell membrane functions [5]. High levels of MDA and low levels of GSH indicate the presence of oxidative stress and have been related to several diseases such as Parkinson’s disease, Alzheimer’s disease, cystic fibrosis, diabetes, pneumonia, hypothyroidism, atherosclerosis, human immunodeficiency virus (HIV) infection, and cancer [6,7,8]. Thus, the monitoring of both GSH and MDA levels is of great clinical importance. However, their determination is challenging due to their chemical instability, low molecular weight, and lack of chromophore or fluorophore moieties, which complicate their direct detection [9].

Thus, the methods developed in recent decades for the detection of GSH and MDA rely mostly on liquid chromatography methods coupled with mass spectrometry or capillary electrophoresis [10,11,12]. Moreover, due to the redox activity of GSH, several electrochemical methods have been developed based on amperometry, cyclic voltammetry, differential pulse voltammetry, square wave voltammetry, or linear sweep voltammetry [13,14]. Although those methods are reliable and sensitive, they require bulky and expensive instrumentation or tedious sample pretreatment, which may affect the stability of GSH and MDA samples. Furthermore, GSH concentration varies from several mM in mammalian cells to several μM in biological fluids, such as blood, urine, and saliva, while MDA concentration in healthy individuals is lower than 1 μM [9,15]. For this reason, methods combining low detection limits with a wide dynamic range are needed for both GSH and MDA determination.

For this purpose, several spectroscopic techniques based on quantum dots, molecular beacons, gold nanoparticles conjugated with small molecules, polymers, and biomacromolecules, as well as fluorescent dyes, such as cyanine, fluorescein, and rhodamine, have been developed [16,17,18,19,20,21], often combined with substrates modified with gold to take advantage of the surface plasmon resonance phenomena for the detection of the targeted analytes [22,23,24]. Among these spectroscopic techniques, Surface Enhanced Raman Spectroscopy (SERS) attracts a lot of interest, as it is a non-invasive technique with minimal background interference and very high sensitivity [25]. The use of noble metals (Au, Ag) as SERS substrates increases detection sensitivity as they provide Raman signal enhancement. In particular, an increase in detection sensitivity up to 10–14 orders of magnitude compared to conventional Raman spectroscopy has been reported when surfaces with metal particle clusters (hot spots) have been used as substrates [26].

Raman signal enhancement by SERS substrates has been attributed to two mechanisms; the electromagnetic and the chemical mechanism. The electromagnetic mechanism arises from the interaction between the optically excited collective electron oscillations (plasmons) and the molecule under study. Coupling between the laser beam and the conductive electrons considerably affects local electromagnetic field distribution in the proximity of the nanostructured metallic surface, increasing the Raman scattering cross-section and, hence, improving the output signal [27,28]. It is known that nanostructures of metals such as Au [29] and Ag [30] can support surface plasmon polaritons. In particular, Ag nanostructures in various shapes have been investigated and have demonstrated significant electromagnetic field enhancement factors [31]. Such structures are suitable for SERS [32,33] and surface-enhanced fluorescence [34]. The influence of plasmonic nanostructures roughness on hot spot intensity distribution plays a key role in SERS. Nanometer-sized gaps between the metals can lead to increased electromagnetic fields and Raman signal enhancement [28]. It is also well documented that Ag gives stronger plasmon resonances compared to other noble materials, such as Au or Cu. There are several reviews covering this in detail [35,36,37]. In particular, for noble metal nanostructures on silicon nanowires, Ag has shown higher signal enhancement factor compared to Au and Au-rich Ag–Au alloys [38,39]. The chemical mechanism is associated with molecular orbital interactions between metal nanostructures, and the signal enhancement comes from three contributions. Firstly, a resonance Raman effect arises due to the incident light matching an electronic transition in the molecule. Secondly, a charge-transfer effect takes place, where the incident light is in resonance with a molecule–metal or metal–molecule transition. Finally, a non-resonant chemical effect occurs due to ground-state orbital overlap between the molecule and the metal [28,32].

Our team has developed SERS substrates using metal-assisted chemical etching of silicon (MACE) with either Ag-aggregates or Ag-dendrites and demonstrated signal enhancement factors in the range of 10^5^ to 10^10^, achieving limits of detection of 10^−13^ M for rhodamine 6G (R6G) [33]. The same structures also proved to be very effective in surface-enhanced fluorescence, enabling the detection of R6G at concentrations down to 10^−12^ M [34]. Thus, these 3-dimensional silver nanostructures show great efficiency for realizing electromagnetic field enhancement and can be useful in biosensing [40,41]. For the substrates implemented in the current work, previously reported finite element calculations [30] have demonstrated that the optical response of Ag-nanoparticles on Si wires supports a particle mode that can be tuned by the Ag cap size and can be hybridized by leaning of Si pillars. Narrow gap sizes between metallic parts lead to “hot spots” that induce strong field enhancement [34]. Bimetallic Au–Ag core–shell nanostructures, and dendrites [38,42] have also attracted some attention since they have shown improved SERS and fluorescence signal detection compared to single-metal Au or Ag structures [35,38]. The position of the plasmon resonance was controlled through the alloy composition to custom-design substrates that match the Raman excitation or the dye emission band. Generally, the plasmon resonance red-shifts with increasing Au concentration in Ag–Au alloys, but careful control of the composition is required to avoid spectral broadening and resonance damping. The Raman signal may be further enhanced with the aid of Raman-sensitive molecules, known as Raman reporter molecules, such as 5,5′-dithio-bis(2-nitrobenzoic acid), rhodamine 6G, and crystal violet, enabling the detection of molecules that are very weak Raman scatterers [19].

Despite these developments, up to now, few methods for the detection of GSH with SERS have been reported in the literature, as the enhancement factor for direct detection of GSH is relatively low, and, therefore, the detection sensitivity is also relatively low [43,44]. A substantial increase in sensitivity of direct GSH detection with SERS (from 1 μΜ to 50 nM) has been reported after thermal treatment of colloidal silver nanoparticles with GSH solution to create a dry film on the SERS substrate [45]. However, most methods rely on indirect detection schemes [46,47,48]. More specifically, the SERS substrates were modified with 5,5′-dithio-bis(2-nitrobenzoic acid), which reacts with the thiol group in GSH, producing the Raman active compound 2-nitro-5-thiobenziate and achieving a detection limit of 50 nM [47,48,49]. Another approach to the indirect detection of GSH is based on the replacement of a Raman reporter molecule, such as rhodamine 6G (R6G), 4-mercaptopyridine, or crystal violet adsorbed onto the SERS substrate from GSH molecules due to the higher affinity between thiol groups and silver nanoparticles [50,51,52]. This method also leads to a detection limit of 50 nM [51,52].

Regarding MDA determination with SERS, there are only three reports in the literature, which are based on derivatization of MDA with either 2-thiobarbituric acid [53,54] or 4-aminophenylthiophenol [55], achieving detection limits of 3.2 nM and 50 nM, respectively.

In this work, silicon nanowires decorated with silver nanoparticles made by metal-assisted chemical etching (MACE) were evaluated as substrates for the determination of GSH and MDA in both water solutions and artificial and real saliva samples. The substrates implemented have been shown to significantly enhance the signal of directly adsorbed Raman reporters such as rhodamine 6G [33,34], but have not so far been employed for the detection of biomolecules in aqueous or other samples. For GSH determination, both direct detection and detection after substrate modification with crystal violet were investigated, while for MDA, derivatization with 2-thiobarbituric acid prior to incubation with the substrate was applied. Several assays’ parameters were optimized using aqueous solutions of GSH and MDA prior to testing with solutions of both analytes prepared in artificial saliva [56] spiked with known concentrations of both analytes or real saliva samples. Saliva testing offers several advantages compared to blood and urine testing due to the ease and non-invasive method of sample collection through swabs or using properly designed containers. In addition, especially for stress markers, there are well-established correlations between their levels in the blood and in the saliva [57]. However, saliva is a complex matrix, containing several inorganic ions, organic molecules, enzymes, and hormones, and its composition varies both between individuals as well as in the same individual over the duration of a day [57]. Thus, its presence is expected to considerably affect the SERS signal and therefore the detection sensitivity of targeted analytes.

## 2. Materials and Methods

### 2.1. Materials

Hydrofluoric acid (HF) was purchased from Technic Inc. (Saint-Denis, France). Nitric acid (HNO_3_) came from Lach-Ner Ltd. (Neratovice, Czech Republic). Silver nitrate (AgNO_3_), reduced L-glutathione, 2-thiobarbituric acid, and malondialdehyde tetrabutylammonium salt were purchased from Sigma-Aldrich (Taufkirchen, Germany). Crystal violet (CV) was obtained from Acros Organics (Geel, Belgium). Salivette^®^ synthetic swabs came from Sarstedt (Numbrecht, Germany). The water used throughout the study was distilled.

### 2.2. Preparation of SERS Substrates

The SERS substrates were fabricated, as described in previous work [33,34], by metal-assisted chemical etching (MACE) to form silicon nanowires (SiNWs) decorated with silver nanoparticles on silicon substrates. Briefly, p-type (100)-oriented monocrystalline silicon wafers were firstly immersed into an aqueous solution containing 0.02 M AgNO_3_ and 4.8 M HF for 6 min to fabricate the SiNWs. Then, the substrates were dipped in 50% v/v HNO_3_ for 4 min in order to dissolve the silver nanostructures formed during the etching process. Finally, the wafers were re-immersed for 7 s in a solution containing 0.02 M AgNO_3_ and 4.8 M HF, resulting in the formation of silver aggregates mainly on the top of the SiNWs tips. The SERS substrates prepared following this process were characterized by scanning electron microscopy (SEM) using a JSM- 7401F SEM instrument (JEOL Europe bv; Zaventem, Belgium) working at 30 kV.

### 2.3. Glutathione Detection Protocol

For the detection of glutathione, SERS substrates with dimensions of 0.5 × 0.5 cm^2^ were firstly immersed into 300 μL of a 3 μg/mL aqueous CV solution for 30 min. Then, the substrates were washed twice with distilled water, dried, and incubated for another 30 min with 300 μL of glutathione solutions at concentrations ranging from 0 to 100 μM. Finally, the substrates were washed with distilled water, left to dry, and then measured by micro-Raman spectroscopy using an EnSpectr RamMics M532 Raman spectrometer coupled with an Olympus BX43 microscope and a 532 nm laser diode as an excitation source. The laser beam was focused onto the samples to a spot size of around 2 μm through a 20× objective lens. The laser power density was kept below 0.1 mW/μm^2^, and the integration time was kept to 1 s to avoid local heating effects.

### 2.4. Malondialdehyde Detection Protocol

For malondialdehyde determination, derivatization with 2-thiobarbituric acid (TBA) was performed in a strongly acidic solution. In brief, a 42 mM aqueous solution of TBA was prepared by dissolving 0.3025 g of TBA in 50 mL of distilled water and heating at 55 °C for 45 min. When the TBA solution was cooled to room temperature, 500 μL of aqueous MDA solutions (with concentrations ranging from 0 to 3.2 μM) were mixed with 750 μL of a 440 mM H_3_PO_4_ solution and 250 μL of the TBA solution and left to react at 90 °C for 1 h. The reaction mixture was cooled to room temperature, and then 300 μL of each mixture was added into wells containing SERS substrates with dimensions of 0.5 × 0.5 cm^2^ and incubated for 20 min at room temperature. SERS measurements were carried out using an inVia Reflex microscope (Renishaw, UK) equipped with a solid-state laser emitting at 785 nm. The laser beam was focused onto the samples down to a spot size of around 1 μm by means of a 50× (NA = 0.75) objective lens on a Leica DMLC microscope. The power density of the laser was kept lower than 0.06 mW/μm^2^ to avoid local heating effects, and the integration time was set to 10 s.

### 2.5. Determination of GSH and MDA in Artificial Saliva and Saliva Samples

To simulate natural saliva, the following solution was prepared according to a protocol from the literature [56]: 0.13 g/L NaCl, 0.96 g/L KCl, 0.66 g/L KH_2_PO_4_, 0.63 g/L NaHCO_3_, 0.19 g/L KSCN, 0.23 g/L CaCl_2_, 0.2 g/L urea, 0.76 g/L Na_2_SO_4_, and 0.18 g/L NH_4_Cl. The pH of the solution was adjusted to 6.8 prior to the addition of GSH and MDA to prepare the calibrators. For the collection of saliva samples, Salivette^®^ cotton swabs were used. The individuals chewed the swabs for 1 min, and then the samples were centrifuged at 1500 rpm for 10 min. The extracted saliva samples were pooled prior to analysis for GSH and MDA determination. The Raman spectra for GSH and MDA calibrators in artificial saliva and saliva samples were received following the protocols described in 2.3 and 2.4, respectively.

## 3. Results and Discussion

### 3.1. Characterization of SERS Substrates

**Figure 1:**
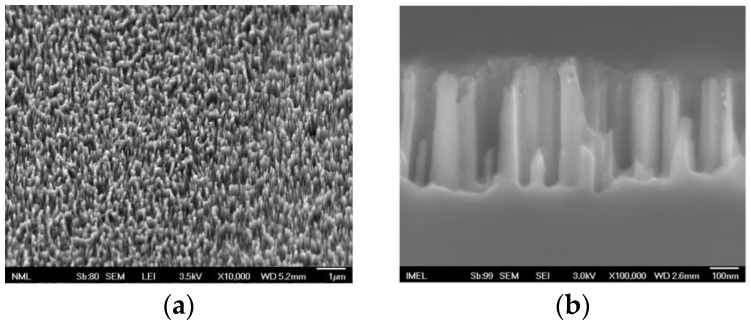

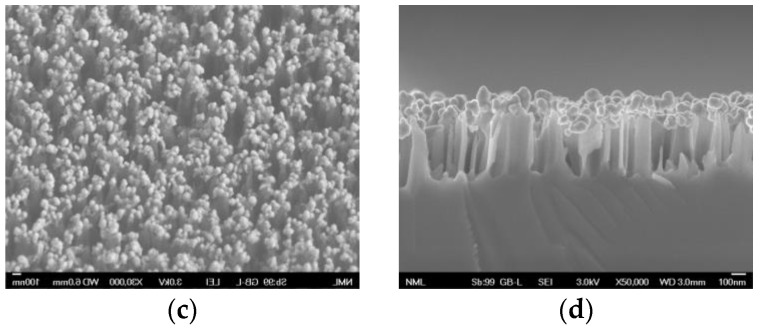
SEM images of SiNWs prior to ((**a**): top view (scale bar 1 μm, magnification ×10,000); (**b**): cross-section (scale bar 100 nm, magnification ×100,000)) and after growth of silver aggregates on the surface of SiNWs ((**c**): top view (scale bar 100 nm, magnification ×30,000); (**d**): cross-section (scale bar 100 nm, magnification ×50,000)). The Ag aggregates grow mainly at the tips of the SiNWs.

The SERS substrates implemented in the current study were prepared in silicon wafers following a three-step MACE method consisting of (a) treatment in an AgNO_3_/HF solution during which silicon was etched and silicon nanowires (SiNWs) were formed with Ag dendrites on top, (b) immersion in HNO_3_ solution to remove the Ag structures, and (c) re-immersion in the AgNO_3_/HF solution to create new Ag nanoparticles. Figure 1 shows top and cross-section SEM images of the SERS substrates prepared following the MACE method. In particular, in Figure 1a,b, SEM images of bare SiNWs are provided, whereas in Figure 1c,d, the respective images after the creation of Ag aggregates are shown. From Figure 1c, it is evident that the surface is characterized by high homogeneity in terms of the density and size of the Ag aggregates formed. In the cross-section SEM image in Figure 1d, the deposition of Ag in the form of aggregates at the tips of the SiNWs is demonstrated without any indication of deposition at the walls or at the bottom of the substrate. The SiNWs have lengths in the range of 440–480 nm, while the silver aggregates size varied between 120–150 nm.

In Figure S1, the UV-vis absorption spectra of bare SiNWs and SiNWs/Ag nanoparticles are presented. The absorption spectra were obtained from the respective diffuse reflection spectra [33,34] by applying the standard Kubelka–Munk method [58]. As shown, the absorption of substrates with bare SiNWs is stronger at wavelengths greater than 350 nm (VIS light), while substrates with Ag nanoparticles on top have significantly higher absorption at UV.

### 3.2. Raman Signal Enhancement Factor of SiNWs/Ag Aggregate Substrates

**Figure 2:**
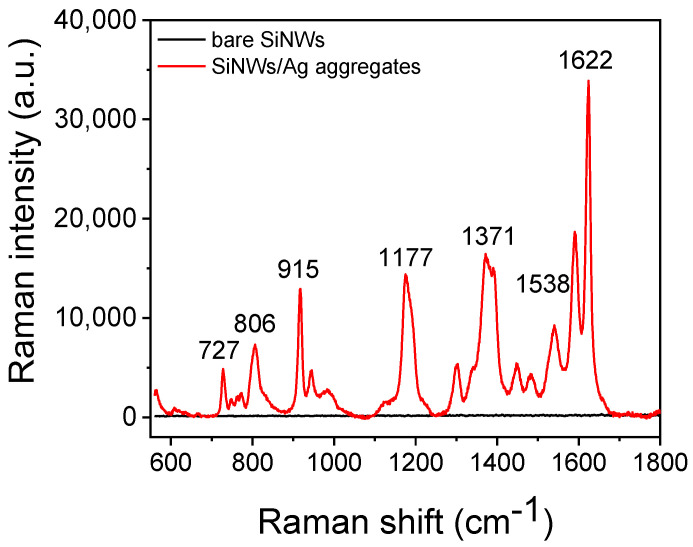
SERS spectra received from surfaces with bare SiNWs (black line) or SiNWs decorated with Ag nanoparticles after incubation with a 30 μg/mL aqueous CV solution using a laser at 532 nm.

In order to evaluate the SiNWs/Ag nanoparticles substrates performance with respect to Raman signal enhancement, the signal received from such surfaces after incubation for 3 h with a 30 μg/mL aqueous solution of CV was compared to that obtained by bare SiNWs. In Figure 2, the SERS spectra of CV obtained from both surfaces are presented. CV exhibits characteristic peaks at 727, 806, 915, 1177, 1371, 1538, and 1622 cm^−1^, which were apparent only on the spectrum received from the surface with SiNWs decorated with Ag nanoparticles. No detectable Raman signal could be traced from the surfaces with bare SiNWs, indicating the extremely high Raman signal enhancement by the Ag nanoparticles.

### 3.3. GSH Determination with SERS

For the determination of GSH in the SiNWs/Ag nanoparticles substrates, two different approaches were investigated: the first is based on direct detection of GSH bound onto the substrates through the reaction of its thiol group with the Ag nanoparticles, and the second onto replacement by GSH (again through binding to the Ag nanoparticles) of CV molecules pre-adsorbed onto the SERS substrates. For the second approach, referred to as the “reversed reporting agent method”, several parameters were optimized prior to its application on the SiNWs/Ag nanoparticle substrates, including the concentration of the CV solution and the incubation duration of this solution with the surface, as well the duration of the subsequent incubation with the GSH solutions.

#### 3.3.1. Optimization of GSH SERS Determination through CV

**Figure 3:**
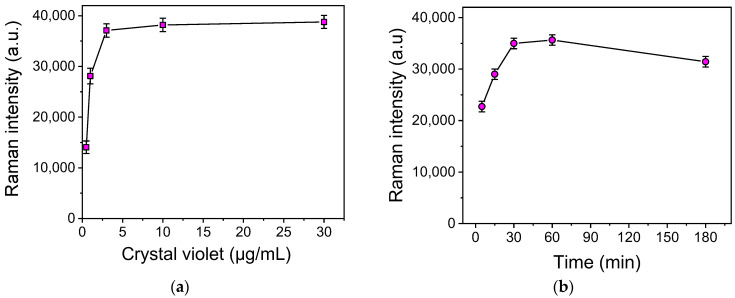
(**a**) Effect of CV concentration on Raman signal intensity at 915 cm^−1^ obtained from the SiNWs/Ag nanoparticles substrates. The substrates were incubated with the CV solutions for 3 h. (**b**) Effect of incubation time with the CV solution on the Raman signal intensity obtained from the SiNWs/Ag nanoparticle substrates. The CV solution concentration was 3 μg/mL. Each point is the mean value of measurements received from 3 substrates (3 different spots per substrate) ± SD.

The first parameter optimized was the concentration of CV in the solution used to modify the SiNWs/Ag nanoparticles’ substrates. For this purpose, aqueous solutions of CV at concentrations of 1, 3, 10, and 30 μg/mL were incubated with the substrates for 3 h, and then the Raman spectra were acquired using a laser at 532 nm. As shown in Figure 3a, the Raman intensity increased as the CV concentration increased, and maximum plateau values were reached for concentrations equal to or higher than 3 μg/mL. Thus, this concentration was selected for further experimentation.

To determine the effect of the time of CV adsorption onto the substrates on the intensity of the Raman signal, a 3 μg/mL aqueous CV solution was incubated with the SiNWs/Ag nanoparticle substrates for 5, 15 30, 60, and 180 min. As depicted in Figure 3b, the maximum Raman signal was obtained for incubation duration between 30 and 60 min, while by prolonging the incubation time up to 3 h, a decrease in the signal was observed. Thus, a 30 min incubation was adopted in the final protocol in order to shorten as much as possible the whole assay time.

**Figure 4:**
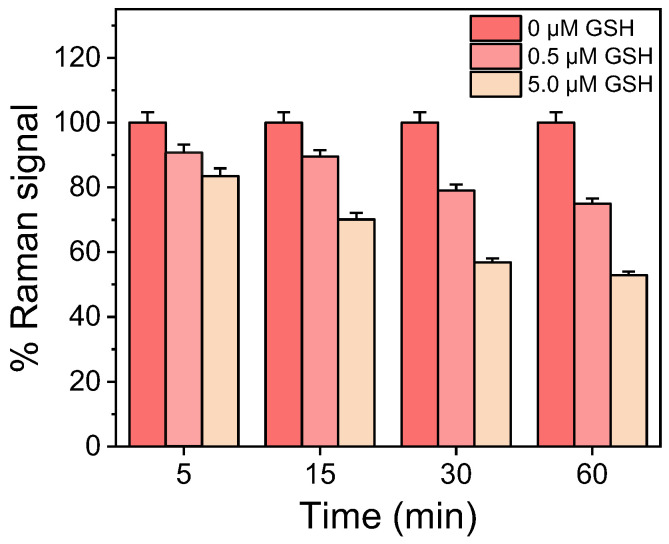
Percent signal obtained from SiNWs/Ag nanoparticles substrates incubated with aqueous GSH solutions with a concentration of 0.5 and 5 μM for 5, 15, 30, and 60 min with respect to the signal obtained in the absence of GSH. All substrates have been modified prior to the incubation with the GSH solutions with a 3 μg/mL CV solution for 30 min. Each point is the mean value of measurements received from 3 substrates (3 different spots per substrate) ± SD.

Once the concentration of CV solution and incubation time with the SiNWs/Ag nanoparticles substrates were established, the incubation time with the GSH solutions was also determined. Therefore, aqueous solutions of GSH at concentrations of 0.5 and 5 μM were incubated with substrates modified with CV for 5, 15, 30, and 60 min. In Figure 4, the percentage signals obtained from surfaces incubated with 0.5 and 5 μM GSH to the signal obtained from surfaces incubated with distilled water (zero GSH calibrator) are presented. As shown, the percentage signal drop with respect to the zero calibrator increased as the incubation time increased up to 30 min. The percentage signal drop regarding the calibrator containing 0.5 μΜ GSH with respect to zero calibrator signal was ~9% for 5 min incubation and reached 21% when the incubation time increased to 30 min. Further increases in the incubation time did not significantly improve the assay sensitivity (the percent signal drop for the 0.5 μΜ GSH solution was 25% after 60 min of incubation). Thus, a 30 min incubation of CV-modified surfaces with the GSH solutions was adopted in the final protocol.

#### 3.3.2. Comparison of Direct and Indirect GSH Determination with SERS

**Figure 5:**
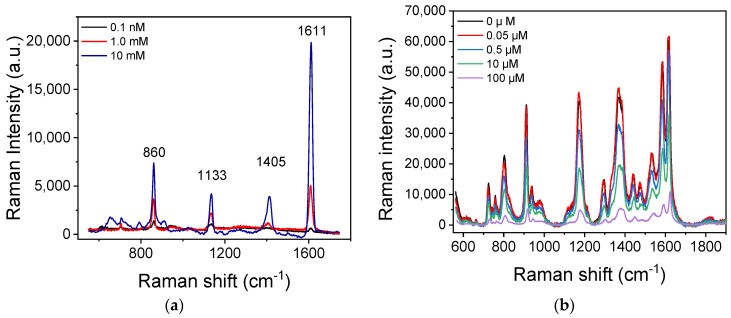

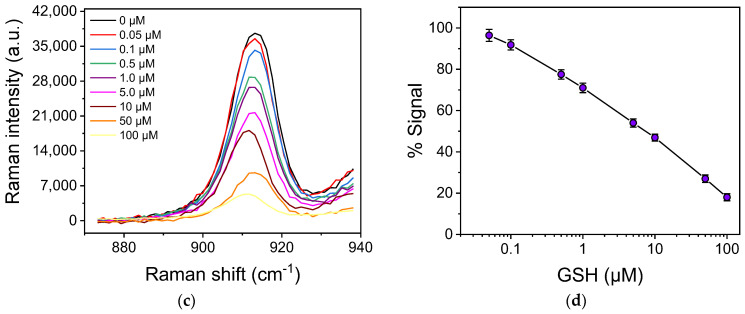
(**a**) Raman spectra from SiNWs/Ag nanoparticle substrates incubated with aqueous GSH solutions of 0.1 (black line), 1.0 (red line), and 10 mM (blue line) concentrations at 785 nm laser excitation. (**b**) Raman spectra from SiNWs/Ag nanoparticles substrates modified with CV prior to the incubation with GSH solutions of 0 (black line), 0.05 (red line), 0.5 (blue line), 10 (green line), and 100 μM (violet line) concentrations at 532 nm laser excitation. (**c**) Partial Raman spectra including the peak at 915 cm^−1^ obtained from SiNWs/Ag nanoparticle substrates modified with CV prior to the incubation with GSH solutions with concentrations ranging from 0 to 100 μΜ. (**d**) Typical calibration curve of GSH. Each point is the mean of measurements received from 5 substrates (3 different spots per substrate) ± SD.

Regarding the direct GSH detection, in Figure 5a, the SERS spectra received from SiNWs/Ag nanoparticles substrates incubated with aqueous solutions of GSH at concentrations of 0.1, 1.0, and 10 mM are presented. As shown, four characteristic peaks were observed at 860, 1133, 1405, and 1611 cm^−1^. All peaks are clearly identified in the spectra from the surfaces incubated with a GSH solution with a concentration higher than 1 mM, whereas in the spectrum obtained from the substrate incubated with a 0.1 mM GSH solution, the peaks can be barely distinguished. Therefore, the concentration of 0.1 mM can be considered as the limit of detection for the direct detection method.

On the other hand, following the optimized protocol for the indirect determination of GSH on substrates modified with CV, the detection sensitivity of GSH was dramatically improved. Ιn Figure 5b, the spectra received from SiNWs/Ag nanoparticle substrates modified with CV prior to the incubation with GSH calibrators with concentrations ranging from 0 to 100 μΜ are presented. In this case, the signals corresponding to Raman spectrum peaks of CV at 727, 806, 915, 1177, 1371, 1538, and 1622 cm^−1^ were reduced inversely proportional with the GSH concentration. Amongst the characteristic peaks of CV, the intensity of the peak at 915 cm^−1^ was selected for the creation of the GSH calibration curve, and this particular spectral range is presented in Figure 5c. The selection of the peak was based on the fact that its intensity showed a higher percentage change with respect to the changes observed for the other peaks when the GSH concentration changed. Taking into account the Raman signal value at the peak, the percentage signal drop that corresponded to the different concentrations of GSH with respect to the signal obtained from surfaces that reacted with the zero calibrator (i.e., in absence of GSH) was calculated and plotted against the GSH concentration in the calibrators. A typical calibration curve is depicted in Figure 5d. The limit of detection was calculated as the GSH concentration that corresponded to a percent value equal to 100–3SD of 10 repetitive measurements of the zero calibrator, and it was 50 nM. Thus, compared to the method of GSH detection through its direct incubation of the SiNWs/Ag nanoparticles substrates, an enhancement factor of at least 10^3^ was achieved regarding the assay’s analytical sensitivity.

### 3.4. MDA Determination with SERS

**Figure 6:**
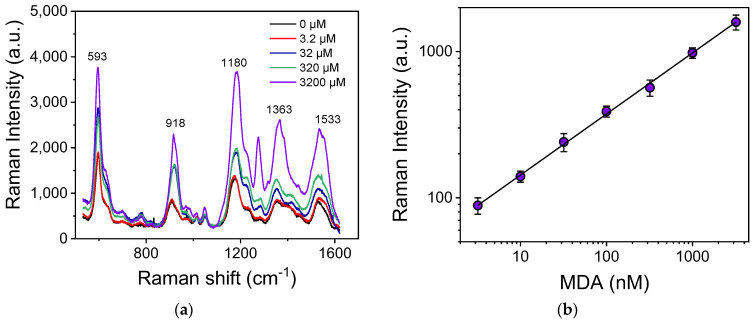
(**a**) SERS spectra of TBA-MDA derivative for MDA concentrations ranging from 0 to 3200 nM. (**b**) Calibration curve of MDA. Each point corresponds to measurements received from 5 substrates (3 different spots per substrate) ± SD.

MDA determination was based on its derivatization with TBA. TBA exhibits typical peaks at 620, 927, 1170, 1327, and 1523 cm^−1^, as shown in Figure S2, where the Raman spectrum of an aqueous TBA solution is depicted. In Figure 6a, the SERS spectra of the TBA-MDA derivative corresponding to different MDA concentrations are presented. In all calibrators, the final concentrations of TBA and H_3_PO_4_ were 7 mM and 220 mM, respectively. As indicated in the graph, the peaks in the spectra of the TBA-MDA derivative were shifted compared to that of TBA (Figure S2). More specifically, in the TBA-MDA-derivative spectra, the main peaks appear at 593, 918, 1180, 1363, and 1533 cm^−1^. Although the Raman signal intensity corresponding to each of the five peaks increased as the MDA concentration in the solution increased, the calibration curve was constructed by selecting the characteristic peak at 1533 cm^−1^, which shows the most prominent intensity change with respect to the MDA concentration change. A typical calibration curve is presented in Figure 6b. From this curve, the detection limit was determined at 3.2 nM, while the linear working range extended up to 3.2 μM.

### 3.5. Determination of GSH and MDA in Natural and Synthetic Saliva

**Figure 7:**
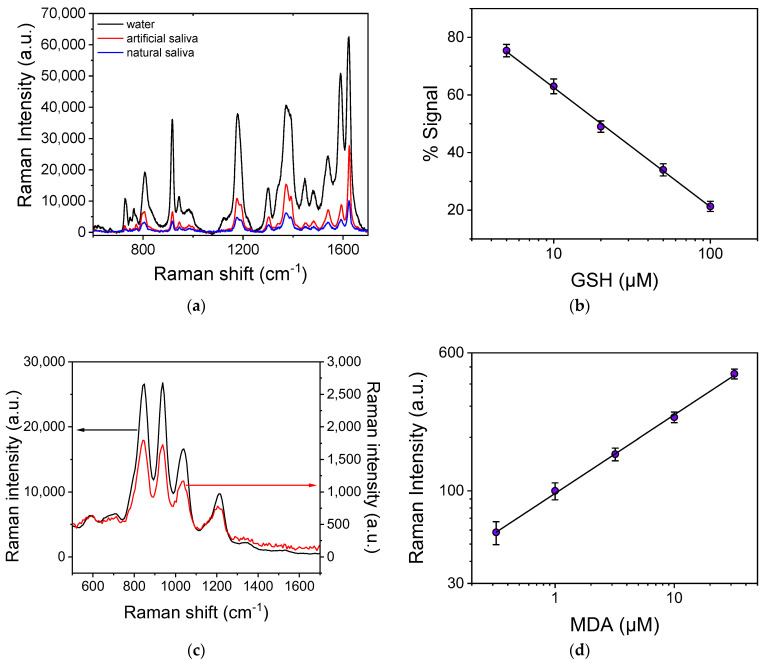
(**a**) Raman spectra obtained from SiNWs/Ag nanoparticle substrates modified with CV and then reacted with a zero-GSH calibrator prepared in water (black line) or artificial saliva (red line), as well as with a natural saliva sample (blue line). (**b**) GSH calibration curve obtained with calibrators prepared in artificial saliva. Each point corresponds to measurements obtained from 5 substrates (3 different spots per substrate) ± SD. (**c**) Raman spectra obtained from SiNWs/Ag nanoparticles substrates reacted with a 32 μΜ MDA calibrator prepared in water (black line) or artificial saliva (red line). The arrows indicate which y-axis corresponds to each spectrum. (**d**) MDA calibration curve obtained with calibrators prepared in artificial saliva. Each point corresponds to measurements obtained from 5 substrates (3 different spots per substrate) ± SD.

Based on the results obtained for the determination of GSH and MDA in water, the next step was their detection in artificial and natural saliva. In Figure 7a, the SERS spectra obtained from surfaces modified with CV and then reacted with a zero-GSH calibrator prepared in water or artificial saliva, as well as with a natural saliva sample, are depicted. As shown, the Raman intensity decreased by approximately five times when the GSH solution was prepared in artificial saliva instead of distilled water. The signal reduction may be attributed in the presence of inorganic compounds containing sulfur (KSCN, Na_2_SO_4_) in the artificial saliva that could react with the substrate replacing CV molecules [59,60,61]. Despite this considerable signal reduction, there was a concentration-related response when the surfaces were reacted with GSH calibrators with concentrations ranging from 5 to 100 μM prepared in artificial saliva (Figure 7b), resulting in a detection limit of 2.0 μΜ. From the calibration curve depicted in Figure 7b, a GSH concentration of 20 μΜ was calculated for the natural saliva sample. This result can be attributed to two sources, the saliva endogenous GSH and additional effects from other compounds present in the real saliva sample but not included in the artificial saliva’s composition [48,57,62]. In particular, due to the detection principle employed, it is expected that small organic molecules containing N and/or S-like amino acids might interfere; however, it has been demonstrated that amino acids which do not contain S do not affect the signal, whereas the presence of amino acids containing S (methionine, cystine, oxidized glutathione) and even amino acids with free thiol groups such as cysteine and N-acetylcysteine marginally increase the signal (~10%) at concentrations much higher than those expected in biological fluids [51]. This is due to the fact that the GSH has a higher binding affinity toward Ag as compared to other compounds. However, the effect of other factors and especially of the way the sample has been collected and treated needs to be examined in great detail using saliva samples from different donors to conclude on the matrix effect in the method developed.

Similarly, regarding MDA detection, the presence of artificial saliva caused a reduction in the Raman intensity for all characteristic peaks by approximately 15 times as compared to the observed peak intensity for MDA solutions in distilled water, as shown in Figure 7c. Thus, the corresponding MDA calibration curve in artificial saliva (Figure 7d) had a detection limit of 0.32 μΜ and a dynamic range from 1.0 to 32 μM. Regarding the analysis of the natural saliva samples, the spectrum obtained was the same as that received for the zero calibrator in artificial saliva indicating the absence of MDA in the sample analyzed. It is important to mention that the method employed for the detection of MDA in saliva is not affected by the presence of other aldehydes such as formaldehyde, acetaldehyde, butyraldehyde, and benzaldehyde, or other compounds that could react with TBA, such as trans-2-hexenal and pyrimidine, since the Raman spectrum of TBA-derivatives of these compounds is completely different from that of the TBA-MDA derivative [54].

Although the detection limit of both the GSH and MDA assay in saliva was lower than those achieved in water, the dynamic ranges cover the values expected in the absence of oxidative stress that, according to the literature, range from 2.0 to 6.0 μM for GSH [48,62] and from 0–0.8 μM for MDA [63]. In the case of oxidative stress, MDA levels can increase to several μΜ, whereas the GSH concentration is expected to be reduced with respect to normal values. Analysis of more saliva samples and recovery tests with such samples will help to further elucidate the saliva matrix effect on the methods developed.

The sample-to-sample signal variation was also determined by running the same calibrator in five different surfaces and calculating the percentage coefficient of variation (%CV) values. The %CV values for the GSH assay ranged from 1.8 to 3.9% for the whole range of concentration covered by the calibrators, while for the MDA assay, the %CV values ranged from 6.4 to 12.8%.

### 3.6. Comparison with Other Methods

Only a few methods have been reported in the literature for the detection of GSH with SERS either in aqueous solutions or biological fluids, since GSH is considered a Raman-insensitive molecule. In particular, regarding the direct detection of GSH, the methods are characterized by low sensitivity. For example, Kuligowski et al. employed silver nanoparticles, with a diameter ranging from 30 to 40 nm, for GSH determination in whole blood, achieving a detection limit of 13 μM, which is considered low for GSH determination in serum samples [44]. Thermal treatment of GSH with the SERS substrate has been found to significantly increase assay sensitivity, as reported by Huang et al., who developed a “heat-induced SERS sensing method” [45]. In this method, GSH was mixed with silver nanoparticles and heated prior to measurement, enabling GSH detection at concentrations from 50 to 800 nM. Although this method is fast and sensitive, the thermal treatment of GSH may lead to false results due to the thermal decomposition of the molecule. For the development of sensitive assays, indirect methods have been adopted for GSH determination. Li et al. constructed Au–Ag nanobowls functionalized with neocuproine-Cu^II^ (Nc-Cu^II^) for the indirect detection of GSH through oxidation of Cu^II^ to Cu^I^ in the presence of GSH. The detection limit achieved was 0.75 μΜ, and the dynamic range extended up to 100 μΜ [46]. In two other reports the indirect detection of GSH was based on a reaction with dithiobis-(2-nitrobenzoic acid) adsorbed on silver nanoparticles aggregated on silicon porous discs [47] or on self-assembled Au nanocubes [48]. GSH was detected within 30 min at concentrations ranging from 50 to 750 nM [47,48]. A similar mechanism was followed when N-succimidyl-3-(2-pyridythio)propionate (SPDP) was used as the Raman reporter molecule for GSH detection on a silicon substrate modified with silver nanoparticles [64]. The detection limit was 10 nM, and the dynamic range extended up to 500 nM in phosphate-buffered saline and up to 1 μM in serum. The total analysis time was 4 h [64]. GSH was also detected with a group of methods referred to as “reversed reporting agent” methods, which are based on the replacement by GSH of molecules with a strong Raman signal from the substrate. Such molecules include rhodamine 6G, CV, thiacyanine, or 4-mercaptopyridine and have been used along with Fe_3_O_4_/Ag nanoparticles and Au-core/Ag-shell nanoparticles as SERS substrates [50,51,52]. In particular, GSH was detected at concentrations ranging from 1 to 100 μΜ using rhodamine 6G on silver nanoparticles substrate [50], 50 to 700 nM using CV on Fe_3_O_4_/Ag particles [51], and 50 to 150 nM using 4-mercaptopyridine on Au-core/Ag-shell particles [52]. The analysis time varied from 20 to 60 min. The type of Raman reporter molecule used, the sample matrix, the detection limit, and the dynamic range of the SERS literature methods are listed in Table 1, along with the respective data for the method developed in the current work. From the data presented in Table 1, it can be concluded that the method developed using the SiNWs/Ag nanoparticles surfaces as SERS substrates has a detection limit comparable to that of most of the methods in the literature, in combination with a very wide dynamic range. Furthermore, the method is based on a 3D-solid SERS substrate and not in colloidal solutions of noble metal nanoparticles, as is the case for most of the published ones. Regarding the total analysis time, both the incubation of the surface with the CV solution and with the GSH calibrators or the sample could be reduced from 30 to 15 min, leading to a total assay duration of 30 min; this, however, will cause a signal reduction of approximately 35%, and an increase in detection limit from 50 to approximately 100 nM.

Regarding MDA determination with SERS, to the best of our knowledge, only three studies have been reported so far in the literature. More specifically, derivatization of MDA with TBA was employed for the detection of MDA in aqueous solutions, using gold or silver nanoparticles as SERS substrates [53,54]. The detection limit was 3.2 nM, and the dynamic range extended up to 3.2 μM for both methods [53,54]. In a more recent study, TBA was replaced by 4-aminophenylthiophenol and SERS determination was performed on Au@Ag nanoparticles achieving a detection limit of 0.5 μM [55]. The detection limits and the dynamic range are similar to those achieved in the current work, while it is the first time that MDA is detected on a solid substrate through SERS.

No studies have been reported in the literature, so far, for the detection of GSH and MDA in saliva samples with SERS. Regarding GSH, the methods reported so far are based on chromogenic reactions or enzymatic assays with detection limits varying from 10 to 90 μM [64,65]. Concerning MDA, photometric methods relying on the determination of the colored product formed by the reaction of MDA with TBA have been reported with detection limits from 0.02 to 3 μM [63,65,66]. In addition, a commercially available competitive enzyme immunoassay kit involving an anti-MDA polyclonal antibody with a detection limit of 1.33 μM has been used to determine MDA in saliva samples [67].

## 4. Conclusions

In this work, a SERS substrate based on silicon nanowires decorated with silver nanoparticles was evaluated for the determination of GSH and MDA in saliva through SERS. In aqueous solutions, the detection limits of GSH and MDA were 50 nM and 3.2 nM, respectively. In the presence of synthetic saliva, the detection sensitivity was lower and reached 2.0 μM for GSH and 0.32 μM for MDA. Although these detection limits are adequate for the determination of both molecules in saliva samples, a more extensive study regarding the saliva matrix effect on GSH and MDA determination by SERS and comparison with already-established methods for the determination of GSH and MDA in biological samples is required. Once the validity of the results obtained in saliva using the methods developed is established, their application for the determination of GSH and MDA in other biological fluids should be tested with the aim to develop a method that would enable correlation studies of GSH and MDA levels in different biological fluids of a single individual. The facile fabrication of the SERS substrates and the good analytical characteristics of the assays developed for the determination of GSH and MDA paves the way for further exploitation of these SERS substrates in other research areas.

## Figures and Tables

**Table 1:** Comparison of GSH detection methods with SERS.

SERS Substrate/Probe	Probe	Matrix	LOD	Dynamic Range	Time	Ref.
Silver colloids	-	whole blood	13 μM	13–2200 μM	5 min	[44]
AgNPs	-	water	50 nM	100–800 nM	-	[45]
Au-Ag nanobowls	Nc-Cu	water	250 nM	0.75–100 μM	-	[46]
PSDs/AgNPs	DTNB	water/serum	75 nM	50–570 nM	30 min	[47]
Au nanocubes	DTNB	PBS/serum	50 nM	50–750 nM	30 min	[48]
AgNP monolayer film	SPDP	PBS/serum	10 nM	10–500 nM	4 h	[62]
AgNPs	rhodamine 6G	water	1 μM	1–100 μM	20 min	[50]
Fe_3_O_4_/Ag NPs	CV	water/whole blood/cell lines	40 nM	50–700 nM	35 min	[51]
Au-core/Ag-shell NPs	4-mercapto-pyridine	water	50 nM	50–150 nM	60 min	[52]
SiNWs/Ag NPs	CV	water/artificial saliva/saliva	50 nM	0.10–100 μM	60 min	this work

## Data Availability

The data presented in this study are available on request from the corresponding author. The data are not publicly available due to privacy issues.

## Supplementary Materials

The following supporting information can be downloaded at: https://www.mdpi.com/article/10.3390/bios13020273/s1, Figure S1: Absorption spectra from the diffuse reflection spectra by standard Kubelka–Munk Function: F(R) = k/s of bare SiNWs (full line) and SiNWs decorated with Ag nanoparticles (dashed line), where k is the molar absorption coefficient and s the scattering factor. Figure S2: SERS spectrum of a 42 mM aqueous TBA solution.
