# Supplementary material for: SERS Determination of Oxidative Stress Markers in Saliva Using Substrates with Silver Nanoparticle-Decorated Silicon Nanowires
Source: Biosensors (Basel). 2023 Feb 14;13(2):273. doi: 10.3390/bios13020273 (PMC9953924; doi:10.3390/bios13020273)
Supplement: Supplementary file 1 [file biosensors-13-00273-s001.zip › biosensors-2178095-supplementary.pdf]

biosensors

Supplementary Materials

SERS Determination of Oxidative Stress Markers in Saliva Using Substrates with Silver Nanoparticle-Decorated Silicon Nanowires

Anastasia Kanioura[1], Georgia Geka[1], Ioannis Kochylas[2], Vlassis Likodimos[2], Spiros Gardelis[2], Anastasios Dimitriou[3], Nikolaos Papanikolaou[3], Sotirios Kakabakos[1] and Panagiota Petrou[1,*]

[1] Immunoassays/Immunosensors Lab, Institute of Nuclear & Radiological Sciences & Technology, Energy & Safety, NCSR "Demokritos" Aghia Paraskevi 15341, Greece

[2] Section of Condensed Matter Physics, Department of Physics, National and Kapodistrian University of Athens, Zografou 15771, Greece

[3] Institute of Nanoscience & Nanotechnology, NCSR "Demokritos", Aghia Paraskevi 15341, Greece

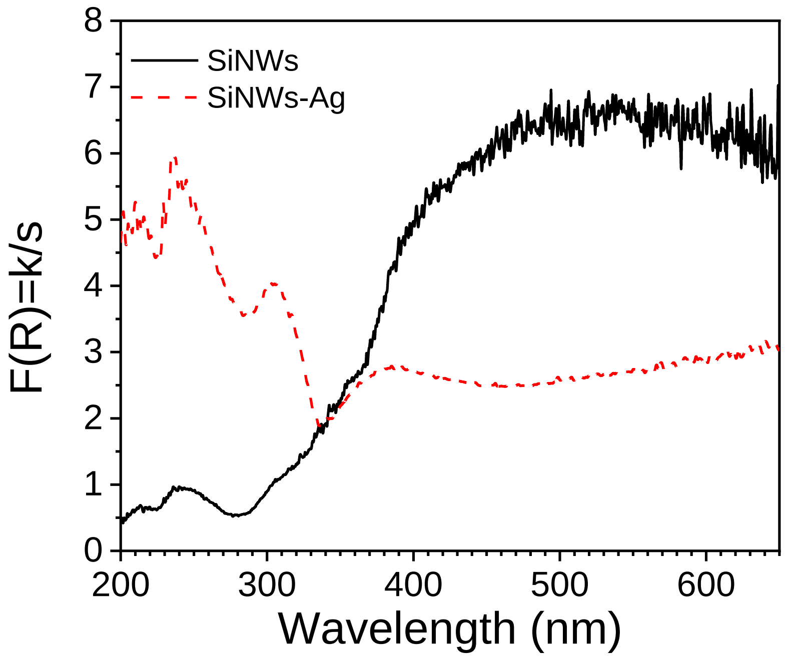

Figure S1. Absorption spectra from the diffuse reflection spectra by standard Kubelka-Munk Function F(R) = k/s of plain SiNWs (full line) and SiNWs decorated with Ag nanoparticles (dashed line); where k is the molar absorption coefficient, and s the scattering factor.

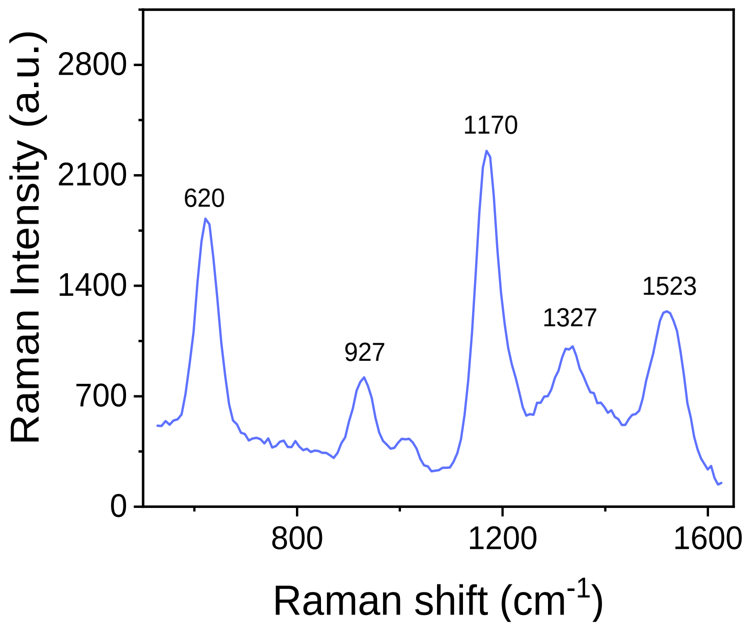

Figure S2. SERS spectrum of 42 mM aqueous TBA solution.
